# A qualitative content analysis study on Swedish school nurses’ experiences of meeting students with gender dysphoria

**DOI:** 10.1186/s12912-026-04801-x

**Published:** 2026-05-26

**Authors:** Malin Stenborg, Lisa Örnerfors, Lina Emmesjö, Marie Wilhsson

**Affiliations:** 1 School of Health Sciences, University of Skövde, Skövde, Sweden; 2 Institute of Health and Care Sciences, Gothenburg University, Box 100, Göteborg, 405 30 Sweden; 3 Krokoms Municipality, Krokom, Sweden; 4 City of Gothenburg, Gothenburg, Sweden

**Keywords:** School nurses, Gender dysphoria, Transgender persons, Health, LGBTQ

## Abstract

**Background:**

Adolescents who experience gender dysphoria are exposed to harassment, discrimination, and violence in the school environment to a greater extent than other students. The school nurses play a key professional role in supporting the health of these vulnerable students.

**Methods:**

A qualitative descriptive design. Semi-structured interviews were conducted with eight school nurses in Sweden and analysed via qualitative content analysis.

**Results:**

School nurses perceived students with gender dysphoria to struggle with anxiety, depression, and self-harm. Some school nurses reported an open school climate, while others expressed the opposite. School nurses express it as vital to establish a relationship to instil courage, strengthen and provide support. They strive to be accessible, signal openness, show understanding, and listen. School nurses call for a collective effort from school staff to elevate competence. They believe that there is a lack of education and directives from management on how school staff should work with students with gender dysphoria.

**Conclusions:**

The school nurses highlight a connection between gender dysphoria and ill health among students, which school nurses should be aware of to better identify these vulnerable students’ needs through the professional role of the school nurse. The health dialogue and the health forms are a key tool for initiating conversations and providing support. The school nurse plays a role in creating inclusion, such as adapting the school environment. The lack of self-reported competence described by school nurses, can be overcome by openness regarding the topic.

**Clinical trial number:**

Not applicable.

**Supplementary information:**

The online version contains supplementary material available at 10.1186/s12912-026-04801-x.

## Background

Gender dysphoria in adolescents (persons in the ages 10–19 [1]) has increased as a diagnosis in the 21^st^ century. The increase has been seen in several international studies in the western world; such as in the US2002-2013 [2], Ireland between 2005 and 2014 [3], Canada and Amsterdam 2006–2013 [4], and Norway from 2000 to 2022 [5]. The increasing numbers have especially been found in adolescents assigned female at birth [2–4]. Gender dysphoria is clinical diagnosis, defined as a psychological suffering caused by an individual’s gender identity not matching the sex assigned to the person at birth [6]. Adolescents with gender dysphoria often identify as transgender. The term stands in opposition with the term cis-gender, referring to persons who identify with the gender identity they were assigned at birth [7].

Persons experiencing gender dysphoria have been argued in previous Nordic and US research to self-rate higher prevalence of mental ill-health than cis-gender persons [8–11]. In a study with parents of adolescents with gender dysphoria, the parents described how the majority of their children had been diagnosed with another mental health disorder prior to the onset of their gender dysphoria. The parents also perceived their child to distrust non-transgender persons, trying to isolate themselves from family and non-transgender friends [12]. According to another study, transgender children, adolescents, and their parents expressed that living in line with one’s gender identity and receiving social support and gender-affirming care can foster a positive sense of identity. Previous research from the UK and Australia shows that these factors help individuals feel affirmed in who they are [13, 14]. In contrast, one systematic review found no difference in mental health outcomes for adolescents who had socially transitioned gender [15]. Another review reported limited or inconsistent evidence regarding associations between gender dysphoria, psychological health and body satisfaction [16]. Systematic reviews have also showed uncertainties in puberty blockers effect on gender dysphoria [16–20]. The quality of studies currently available have also been argued to be difficult to evaluated due to being too small in number [21]. According to researchers in commentary, the complexity of especially rapidly or recent onset gender dysphoria underscores the relevance of capturing the multifaceted nature of, and the interplay between, comorbidity [22], social influences, identity development and gender dysphoria [23].

Support from friends, family, and a safe source of support in school can improve health for these adolescents. The support lessens the risk of depression and suicide according to a scoping review on high school staff [24]. Several Swedish reports since 2015 and onward [6, 25–27] has expressed how transgender adolescents attending school are especially exposed to harassment, bullying, discrimination, and violence in the school environment. Similar findings have also been seen in international reports from the US [28] and the UK, with higher school absence and lowered school results compared to cisgender students [29]. Awareness, openness, and inclusion of LGBTQ+ (lesbian, gay, bisexual, transgender, and queer) students have been expressed in previous research and reports as missing in the school environment, as well as among school nurses. In a study conducted in Finland from a junior high student perspective, school nurses were expressed as being uncomfortable with, and avoiding the subject of, gender identity. The adolescents wanted the school nurses to be open, accepting, and honest about their own lack of competence regarding LGBTQ+ identities. They requested information from the school nurse about sexual and gender variety, rather than the information that is solely cis- and heteronormative [30]. Continuous education for school nurses on transgender adolescents has been expressed as being paramount in improving health for transgender students according to a scoping review with high school staff [24], a report in UK with a student perspective [29], qualitative research with school nurses in the US [31], and in Sweden [32], and school nurses and school physicians in the US [33].

In Sweden, as in many other countries, professionals working in healthcare, school, and social work have a legislative requirement, according to the Convention on the Rights of the Child. The Convention on the Rights of the Child states that all children have the right to life, survival, development, and to their identity [34]. School health service in Sweden includes school physicians, school nurses, psychologists, school counsellors, guidance counsellors, and special education teachers. These professionals provide medical, psychological, psychosocial, and special education services to support environments that promote students’ learning, development, and health [35]. Schools, including school health, are required to investigate incidents of discrimination and harassment, according to the Swedish Discrimination Act [36]. The school nurse’s work is to promote students’ health, academic achievement, and development as well as to prevent and identify discrimination among students [37]. From primary school to upper secondary school, all students in Sweden are offered health dialogues with a school nurse as part of schools’ health promotion and prevention work [35]. On July 1, 2025, a new law was enacted in Sweden concerning the formal recognition of gender change and the regulation of gender-affirmative surgery. The objective of this legislation is to facilitate and enhance access to care and support for individuals experiencing gender dysphoria [38]. The National Board of Health and Welfare in Sweden has created a Guideline for Care of Children and Adolescents with Gender Dysphoria [6], which highlights how care for children and adolescents with gender dysphoria is constrained by limited accessibility and insufficient evidence on treatment outcomes. These constraints lead to recommendations that medical interventions should be used only in exceptional cases and within research settings, underscoring the need for improved documentation and follow-up.

Few studies have focused on the perspectives of school nurses. Two research studies from different parts of the US have been found. In these studies, nurse practitioners working in schools expressed the importance of having knowledge about transgender students’ additional risk of ill-health and actively trying to prevent it [31, 39]. The nurse practitioners working in schools expressed having to push aside their personal opinions about the student’s gender identity, and to treat the transgender students in the same way as the other students [40]. The nurses described how students, as well as students’ parents, needed relevant information [31]. One study with school nurses has been conducted previously in Sweden [32], showing school nurses a deep wish to care for a group of adolescents who were seen as suffering. The study also showed insecurities to work with transgender students due to lack of knowledge. The increasing number of adolescents experiencing gender dysphoria, both nationally and internationally, highlights a need for further knowledge. Previous research also points to shortcomings in health-promoting efforts among students and school staff.

In summary, there is limited knowledge about how school nurses experience supporting students with gender dysphoria. Although research highlights these students’ mental health challenges and the importance of school support, evidence is inconsistent. Few studies have explored school nurses’ perspectives. This study addresses this gap by exploring school nurses’ experiences to identify key challenges, opportunities, and support needs in practice. The aim was to describe school nurses’ experiences of interacting with students with gender dysphoria.

## Method

### Design

An inductive descriptive qualitative design was used, which is relevant to understand and explore a phenomenon [41, 42]. The study consists of qualitative interviews with eight school nurses working in school health services. The Consolidated Criteria for Reporting Qualitative Research (COREQ) checklist was used to assure quality [43].

### Inclusion criteria and sample

School nurses with experience in working directly with students with gender dysphoria were eligible for the study. School nurses in Sweden who were actively working as a school nurse could participate. The inclusion criteria of the present study includes that the school nurse had at least one prior meeting with at least one student who experienced gender dysphoria. There was no specification on which specific health situations the school nurses had experience of. Nine school nurses from six regions in Sweden spanning from north to south (Skåne, Västra Götaland, Dalarna, Uppland, Hälsingland, Jämtland) participated in interviews. However, one participant was excluded due to a lack of direct interaction with students with gender dysphoria, leaving interviews with eight school nurses for analysis. They worked in schools with varying socio-economic backgrounds, in primary schools (ages 6–16) and high schools (16–19). The nurses’ years of experience ranged from 3 to 12 years (mean = 6.5), and their ages ranged from 38 to 64 years (mean = 49). Professional qualifications included district nurse, paediatric nurse, and midwife. The authors approached school nurses in their local area who they had no previous relation. The possible participants were contacted to through their work email and asked if they were interested in participating in a study about meeting students with gender dysphoria. If they agreed, the possible participants received another email with an information letter. The information letter described the purpose of the study, information about the authors and the ethical considerations. If a school nurse agreed, an interview time was set. Recruitment followed a convenience sample, where the participants were selected based on their accessibility. The recruitment also included snowball sampling [44], where the existing participants referred to possible additional participants. When another school nurse was suggested, the authors reached out to the suggested participants through their work email and then followed the same form as described above. Written consent was obtained from both school nurses and their supervisors through a signed document at the time of the interview.

### Data collection

The interview followed a semi-structured interview guide which was created for this study (Supplementary file 1). The interview questions were created after a literary review of previous research and through a discussion in the research group. Interviews began with an orientation phase to clarify the study aim, followed by demographic questions to establish context and ease. To explore the school nurses’ experiences, the interviews started with the question “What comes to mind when you hear the term gender dysphoria?” The question was followed by “Describe your experiences working with students with gender dysphoria.” The semi-structured nature of the interviews allowed for flexibility, with follow-up questions based on participants’ responses. Pilot interviews were conducted to refine the questions and ensure they addressed the study’s aim. Based on the pilot interviews, some questions were removed or combined for clarity, and new questions addressing challenges in working with students with gender dysphoria were added. The questions “What knowledge do you have about gender dysphoria and transpersons?” and “How do you experience the well-being of students with gender dysphoria?” was removed. The interviews were conducted in a quiet, undisturbed setting. They were either in person or via video call. The interviews, regardless of being in person or digital, were at the school nurse’s office. If the interview was held through a video call, the authors interviewing was at their home in a quiet secluded space. The interviews lasted between 32 and 61 minutes and were all held in February and March 2024 by the first and second author. Video interviews increase the opportunity for long-distance participation [45, 46], and has been argued to be compatible with in-person interviews in terms of quality of data [47]. However, technical difficulties may arise which may influence the quality of the data during digital interviews [45], but were not experienced in the present study.

### Data analysis

Interviews were transcribed immediately after the interview by the first and second author, to capture nuances in the interviews. These transcripts were read by the entire research group. A qualitative content analysis on the manifest level was used in accordance with Graneheim and Lundman [48], where the explicitly expressed content of the text was analysed. The analysis started by the first and second author identifying domains (larger text segments) related to the study aim. From these domains, meaning units (shorter phrases or words) were extracted by the first and second author by copying them from the transcribed interview text into a table (Table 1). This process was conducted individually, where the author who had not performed the interview picked out meaning units which answered the study aim and condensed them by shortening them while trying to keep their core meaning. The meaning units and condensed version of them were then read by the author who had performed the interview, and a discussion was held to achieve consensus on the condensed version. Next, all authors read through the meaning units and condensed versions. Following this, the first and second authors sat together and coded the condensed meaning units. After this, a discussion was held in the entire research group about the codes. The codes were grouped into sub-categories reflecting shared characteristics by the first, second and third. The sub-categories were grouped together to create main categories. These were then discussed in the entire research group until consensus was reached on category content and names. Disagreements were reached through a discussion to reach consensus on the category names. The categories were distinct, although some overlap was unavoidable, as per the description of the analysis method [48].

**Table 1:** Example of the analysis process

Meaning unit	Condensed	Code	Sub-category	Category
the most important thing is to listen … to understand what the student needs … what advice to give or guide them further	Important to listen to be able to guide	Support	The function of support and guiding	The school nurse’s health promotion work

### Ethical considerations

This study adhered to the ethical principles outlined in the Declaration of Helsinki [49], including respect for autonomy, beneficence, non-maleficence, and justice. The study was designed, planned and performed as per Swedish law [50], and as the study did not involve processing of sensitive personal data, as defined by the General Data Protection Regulation (GDPR) [51], nor did it pose any physical or psychological risks to participants [50], ethical approval from a formal ethics committee was not required. However, ethical discussions and choices were held in the research group. Participants were provided with written information detailing the voluntary nature of their participation and their right to withdraw from the study at any time without consequence. Informed written consent was obtained from all participants, ensuring confidentiality throughout the research process. The voluntary nature of participation was expressed both in writing in the information letter as well as verbally at the interviews. Data was kept on password protected server, which only the research group had access to. Only M.S and L.Ö had access to the identity of the participants, to ensure their confidentiality. The participants were not considered vulnerable during the interview, nor in the recruitment, since they were recruited and interviewed by a master student in school nursing who they had no prior relationship with. Participating in an interview about a phenomenon which could be considered sensitive may cause stress for the participants, even though it was not their own gender identity which were discussed. The time, place and medium of interview was therefore up to the participant. No participant was perceived to have experienced discomfort during the recruitment or interviews.

### Strengths and limitations

Credibility, dependability, confirmability, and transferability are aspects of trustworthiness in qualitative research [52]. To achieve credibility, school nurses with experience working with students with gender dysphoria were recruited, since credibility is strengthened by the participants having experiences of the phenomenon. The sample could be considered small, but credibility in the findings cannot be judged by the number of participants [52]. Rather, participants were perceived to have varied and deep experiences, which contributed to the study findings. The participants were also from different geographic areas, both urban and rural, which contributed to a broader perspective. Some of the school nurses had longer careers working as school nurses than others, who had only been school nurses for a short time. All participants had several experiences of the phenomenon. Qualitative research exists in a paradigm of a value-based process, characterized by multiple realities and multifaceted perceptions of a phenomenon [52]. While the participation number could be considered low, the experiences and descriptions of the school nurses were rich and varied and therefore suitable for analysis. The school nurses were from different areas in the country, rural and urban, from different municipalities, from different grades, with different specialist educations and years as nurses and school nurses. Furthermore, there was no goal of reaching saturation or in the data collection. Rather, the approach of Graneheim and Lundman of data richness and relevance to the aim was the goal during the data collection.

During the analysis, the phase of choosing which codes and supporting quotes will be included in each category relates to the dependability of the study. To strengthen the dependability of the study, all authors discussed the steps taken during the research process to reach consensus. Among the research group, there were different experiences in school nursing-three of the researchers have experience in school health services, and one researcher had experience teaching higher education in gender and sexuality. The researchers are the instrument in qualitative studies, since their development of self-awareness is important for promoting thoroughness and integrity in assessing the findings [53]. Bias on the subject was discussed within the research group thorough the analysis process to minimize the influence of the results. Presenting these pre-understandings strengthens the dependability of the study, as does using more than one researcher to analyse the material [52]. To what extent the result can be transferable to another population must be decided by the reader [52]. It is strengthened by the descriptions of the participants and setting in the background and method section. The study results may be transferable to other contexts with a similar system for school health care. Transferability would be greater in contexts which have similar cultural views as Sweden. Confirmability is achieved when credibility, dependability, and transferability are achieved. This is achieved through thorough descriptions in the methods section and the discussion of the study’s strengths and limitations.

## Result

**Fig. 1:**
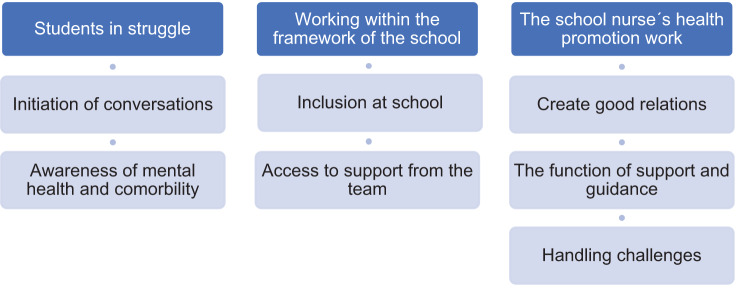
Hierarchy of the results

The analysis resulted in three main categories, which included several subcategories. The hierarchy of these is illustrated in Fig. 1.

### Students in struggle

The school nurses described students with gender dysphoria as students in struggle. The conversation with these students was often initiated by students themselves during individual health dialogues. All adolescents the school nurses met were seen as searching for identity, but it was perceived as especially challenging for students with gender dysphoria. The school nurses were aware that the students with gender dysphoria struggled with anxiety, depression and self-harming behaviours, stomach issues. The school nurses also saw that the students had difficulties with schoolwork and increased absenteeism in school and saw them as students in struggle.

#### Initiation of conversations

The school nurses described different ways in which the initiation of the conversation about gender dysphoria was raised during conversations. The most common approach was when students brough up the topic during health dialogues. The health dialogs were often supported by a survey which the students filled out prior to the health dialog meeting. Some of the school nurses expressed that health surveys with questions about identity invited students to open the conversation about gender identity.

He came in and sat there waiting for the questions about how he feels about his body, knowing that this was where he could get help […] this student was really waiting for that question (School nurse 6).

The school nurses had experiences of students initiating the conversation with them to speak about their gender dysphoria. The visits could be to speak about gender dysphoria directly, or the topic could be brought up when the students sought them out to speak of something else. The school nurses’ descriptions of initiation of conversations with students varied, with students feeling comfortable to open up about the subject in different contexts. School nurses perceived the search for identity as a continuous process for students, particularly during adolescence. The search for identity was perceived by the school nurses to be especially challenging for students with gender dysphoria. The period of adolescence involved a strong focus on fitting in with peers while simultaneously exploring one’s gender identity, according to the school nurses. School nurses observed that students were more likely to initiate a conversation about their gender dysphoria with them when they were a bit older. They perceived this to be because the students found like-minded friends who supported their exploration of gender.

… it’s most common for it to come out in high school. That’s when [the student] feel safe, and maybe [the student] is surrounded by others who are similar to yourself (School nurse 3).

The school nurses expressed how students’ who find like-minded friends seem to contribute to a safer environment for the students to be open in. Additionally, the school nurses experienced that students having gone through puberty could lead to improved emotional stability and increased independence outside of their friend group.

#### Awareness of mental health and comorbidity

The school nurses experienced those students with gender dysphoria frequently struggled with anxiety, depression, and self-harming behaviours. They perceived that as a school nurse they needed an awareness of mental health and comorbidity for these students. Symptoms such as stomach issues, difficulties with schoolwork and increased absenteeism in school was described by the school nurses as early indicators of poor mental health among these students. The degree of gender dysphoria varied among students, impacting the students’ mental well-being to differing extents according to the school nurses. Some students were initially evaluated prior to the school nurses meeting the student by child and adolescent psychiatry. The school nurses described the evaluation to be as to rule out other causes of mental health issues. The evaluation of other mental health issues was perceived by the school nurses as frustrating for the students who were eager to begin their gender dysphoria assessment. In contrast, other school nurses perceive that some students preferred to address their mental health concerns first.

She is incredibly tired of everyone wanting to talk about her gender dysphoria. That’s not what she wants to discuss. She wants to feel good in other aspects first (School nurse 8).

Comorbidity with other mental health diagnoses and neuropsychiatric disabilities (NPF) was common among students with gender dysphoria according to the school nurses. The school nurses highlighted that somatic anxiety was a common experience for the students with gender dysphoria when the biology of their body constantly reminded them of their gender dysphoria. Situations that could increase anxiety, according to the school nurses, was the students seeing their own body when taking a shower, or when they were reminded of bodily functions such as menstruation.

*Anxiety about their body includes concerns such as: ‘Yes, my hips are getting wider, my breasts are developing, and I have soft, feminine body shapes overall’* (School nurse 6).

To alleviate anxiety, school nurses expressed, self-harming behaviours occurred among students with gender dysphoria. The openness of students regarding their self-harm varied, where some openly display their injuries, while others were more reluctant to show their wounds but confided in the school nurse about them. The school nurses expressed it was important to provide information about the risks and complications involved with self-harm.

### To work within the framework of the school

Having competence in LGBTQ+ and about gender dysphoria was needed to create good meetings with these students in school. There were differences in the school nurses’ experiences of working within the framework of the school, where some described an open climate, while others described bullying of students with gender dysphoria. The school nurses did their best to support the students within the framework of the school, and to work out practical solutions for the students. The school nurses found support in their colleges, and some experienced support from the principal, while others expressed the opposite.

#### Inclusion at school

Inclusion at school was a practical work for the school nurses, who described that many students felt secure in expressing a desire for different pronouns, names, or gender attributes than the sex they were assigned at birth. The openness from the students became a bridge to work with the inclusion of them in the school environment. Some school nurses report an open climate at their schools, where students does not experience negative comments or bullying. However, some schools have a culture where comments and insults from peers persist towards students with gender dysphoria.

*There was nothing strange about it really, with his earrings* (School nurse 4).

*The comments in the hallway … there is a lot of insults where people use the word gay* (School nurse 1).

The bullying made it difficult for students with gender dysphoria to be themselves, according to the school nurses. The school nurses strived to improve inclusion at an individual level but also called for a collective effort from all school staff. Sometimes, the school nurses described how their support for students with gender dysphoria included addressing teachers, their co-workers, who misgender students.

*It often gets stuck in discussions; ´it appears in the list, and when I take the rollcall, I see the name listed there, and it cannot be changed in the registration* (School nurse 2).

The school nurses expressed how they too sometimes failed to ask about pronouns or preferred name. The health survey was mentioned by the school nurses as an example where inclusion of students with gender dysphoria into the framework of the school could be improved. The school nurses criticized the health surveys for typically providing only two options for gender identity, while a third option or a text line should be the norm to signal inclusion.

The majority of school nurses believed that changing rooms at the school were not adapted for inclusion of students with gender dysphoria. They often assisted with practical solutions in these cases, such as finding alternative places for students to change. Some schools had gender-neutral changing rooms, but they often come with limitations, such as requiring students to retrieve a key from a teacher or having insufficient shower facilities. In contrast, other school nurses had opposite experiences. An example was a student whose classmates were accepting when the student wished to change in the boys’ changing room instead. Additionally, school nurses described how their schools had revised their approach to puberty discussions in sixth grade to promote inclusion. Previously, classes were divided by gender, but now groups are mixed with consideration for students who do not identify within the traditional binary division.

Another inclusive and norm-critical approach the school nurses described was to incorporate teaching materials that challenge the heteronomy. For example, these materials may address instances when gender identity does not align with biological sex or include same-sex couples in math problems.

#### Access to support from the team

The support that the school nurses had in their work with students experiencing gender dysphoria came mainly from their colleagues in the school nurse group or in school health service team. The school nurses expressed their immediate managers were not their primary source of support. While some of the school nurses experienced support from the school principal, others expressed the opposite. The same discrepancy was found regarding the support of the medically responsible school nurse (MLA) working in the municipality. The school nurses describe that it is important to be able to discuss the topic within the collegial group. To have constructive discussions, the school nurses expressed the need for the school nurse and other professions to feel safe in the relationship with the other professional or in the health team.

*We need to be a work team as well, that you need to feel safe in, to be able to show how ignorant you are and to settle with our own values (School nurse 8).*

According to the school nurses, having competence in the subject of LGBTQ+ and gender dysphoria was a condition to create positive meetings with students with gender dysphoria. Skills-enhancing activities that were mentioned by the school nurses were education, and to discuss the topic at the workplace. Some of the schools were LGBTQ+-certified, according to the school nurses.

### The school nurse’s health promotion work

As part of their health-promoting role, school nurses aimed to support and strengthen all students. In their work with LGBTQ+ students, they sought to signal openness, build trusting relationships, and demonstrate accessibility, which facilitated students’ willingness to discuss gender dysphoria. When needed, nurses collaborated with other healthcare services through referrals. However, working with gender dysphoria was described as challenging, as it often affects students’ overall sense of identity. Because such issues arise infrequently, limited experience and knowledge were perceived as barriers to providing specific support.

#### Create good relations

According to the school nurses, it can take a long time to build a relationship with a student and that following a student for several years benefits the relationship. The experience of some school nurses was that it can be problematic to have health dialogues in the first year of high school, because the school nurse and the student had not had time to get to know each other yet. Other school nurses believed that relationship building began during the first health dialogue. The school nurses experience that there was trust in the profession of school nurse itself, regardless of relationship with the student. Furthermore, the school nurses described that it was important to listen and be empathetic to the student’s narrative.

The school nurses upheld the value of building good relations with their students. One part of that, the school nurses believed was to have prior knowledge of the student’s pronoun. Another way to create good relations was to signal openness around the topic of gender identity. To do this, the school nurses used the rainbow flag symbol. It was described to normalize conversations about LGBTQ+ topics. Furthermore, the school nurses believed that when the topic came up, it was essential not to be judgmental or to react strangely. Rather, the school nurses expressed how it was important to understand and listen to the student.

For the school nurse’s health promotion work as well as to build relations with students, accessibility was as an important aspect for the school nurses. Having an open door created closeness to the students and increased the chances of spontaneous visits by the students, the school nurses experienced. The school nurses also describe that there was an awareness to strategically ask certain questions but avoid others to build relationships. The questions should only concern what the school nurse needed to know in order to help the student, for example which pronoun the student used, the school nurses expressed. Furthermore, the school nurses described that the focus of conversations with students should be on the symptoms of gender dysphoria, while questions about the gender dysphoria itself should be left to specialist care.

*… not asking questions about the physical body. Well, because we’re not interested in that. We are there to help them with how they feel and help them on to the right people and I don’t need to know much (School nurse 5).*

By approaching the student with a focus on the students’ mood, the school nurses perceived that the student sometimes opened up about other areas. The school nurses believed that clear communication and good relations created a security for the student to be open. The school nurses expressed how they in their role should be open about why certain questions were asked. Being explicit about the school nurses reasoning was experienced as especially important when it came to issues such as mental illness and self-harm. They also described that the questions should be asked without paraphrasing, so as to not create uncertainty for the student.

*If you are clear about what you are asking, they can give clear answers back because then I have already set the bar somewhere, how to talk about it (School nurse 6).*

Several school nurses mention that they are honest with the students that they themselves do not know everything and do not have all the answers. Instead, the school nurses aimed to support and listen to the students to create a good relationship.

#### The function of support and guiding

According to the school nurses, part of the school nurse’s health promotion work was to support and guide students by instilling courage, strengthen and provide support. The school nurses feel that the students had a greater need for the school nurse’s support at the beginning of their process regarding gender dysphoria.

*The most important thing is to listen … to understand what the student needs … what advice to give or guide them further …* (School nurse 7).

To support them, the school nurses held counselling with the students about gender dysphoria, relationships or anxiety management. Many of the school nurses had experienced supporting students by guiding them to other health care units when it was needed. The school nurses had experiences of guiding the students to the school counsellor, official referrals to investigate the gender dysphoria at specialist clinics, and to other professions for issues which the school nurses perceived to be related to the gender dysphoria. The school nurses also referred the students to online sources that were deemed by the school nurses to have reliable information. Other initiatives were to refer the students to the youth health care clinic, child and adolescent psychiatry and meeting places for young people within the LGBTQ group.

#### Handling challenges

The school nurses expressed having to handle challenges in working with students with gender dysphoria, since it affected the student’s entire view on their identity. According to the school nurses, it was difficult for them to keep track of concepts and to use the correct pronouns. There was a fear of saying or doing the wrong thing and hurting the students.

*It’s difficult to address these questions, you want to respect the students* (School nurse 3).

The school nurses believed that when this type of question came up so rarely, it became difficult to fully learn or become comfortable with. Too little knowledge and experience made it difficult to give specific advice, according to the school nurses. At the same time, the school nurses believed that it was better to act wrongly, and apologize, than not to dare to act at all.

According to the school nurses, there was a lack of information about gender dysphoria in their education, which made handling these challenges more difficult. The school nurses also believed that there was a lack of directives from management on how school staff should work around these issues. Some of the school nurses expressed how the secrecy around the students’ gender identity made the school nurses feel alone in their knowledge. This loneliness was perceived by the school nurses as difficult when feeling uncertain how to act.

*It can be difficult, as well as being alone in this. In this case with the girl I’ve met, she absolutely didn’t want me to tell anyone (School nurse 5).*

The school nurses felt limited in their work because of this secrecy. If they had been allowed to be open about the student’s issues, the school nurses expressed that they could have explained to other school staff about the reason for the student’s behaviour in school.

## Discussion

The results identify several aspects concerning school nurse’s role in interacting with students experiencing gender dysphoria. The health dialogue and the health survey were identified as important elements in initiating conversation with the students. The importance of the structured health dialogue and the health survey is in line with previous research on school nurses work in Sweden [54, 55], expressing the survey as a structured tool to build dialogue and a relationship with the students. Relationship-building was a central theme in the experiences of the school nurses in the results. The process and importance of relationship building between school nurses and students has also been emphasised in previous research on school nurses in Sweden [55]. The previous research show how relationships is essential to the school nurse’s health promotion work with students, which is the school nurses key role in Swedish schools [37]. This way the school nurses can become a support in the students identity exploration, by promoting enhanced self-awareness and a deeper understanding of identity [56], in the students identity crisis. However, relationship-building was recognised as a process which took time according to the school nurses, highlighting the need for availability, openness and direct communication as important in their interactions with students with gender dysphoria.

Being available for the students also required time, and could be compromised by the increasing workloads for school nurses, a concern raised in previous research on school nurses work [54, 55]. However, time constraints were not explicitly mentioned by the school nurses in the current study. Rather, the school nurses described other challenges in their work with supporting students with gender dysphoria. The school nurses showed openness by using symbols such as the rainbow flag. Previous research on LGBTQ+ persons has shown that the rainbow flag is recognised as a symbol of openness and acceptance and can be an important marker for navigating between safe and unsafe environments. However, the flag must be accompanied by genuine awareness and openness, as the study also provided examples of situations in which the flag was associated with a false sense of security [57]. The school nurses in the current study emphasised the importance of empathy, actively listening to students. In a previous study [58], the school nurse is described as playing a central role as a professional and trustworthy adult with whom students can confide. In the present study, the school nurses expressed how they strived to become this adult. They did so by using correct pronouns and the student’s chosen name to support them in their gender exploration, even if they sometimes failed. These experiences align with what transgender persons themselves have requested from health care personal, and was upheld as respectful and affirming treatment [59, 60]. Being called by ones chosen name and pronounce has been expressed by LGBTQ+ students as crucial for fostering productive discussions regarding identity [30]. The school nurses in the current study experienced that communication was essential, and that their questions primarily focused on the student’s well-being and the symptoms of gender dysphoria, without delving deeply into the underlying causes of the dysphoria itself. Previous research on Swedish school nurses is in line with the school nurses in the precent study’s view [55], noting that school nurses refer students to other services with greater expertise when they feel their own competence is insufficient.

Students with gender dysphoria were described by the school nurses as being engaged in a process of identity exploration, which promoted a role confusion for them. This search for identity is a developmental task typical of adolescence [56], but for these students, it also includes the search for gender identity. By engaging in active listening, the school nurses tried to support the student in navigating their gender exploration, and their other mental health issues. In line with Erikson’s theory [56] of identity development, adolescence is characterized by identity exploration and potential role confusion. Students with gender dysphoria were described by school nurses as being actively engaged in this process, where the exploration of identity also includes gender identity and may intensify role confusion. Through active listening, school nurses sought to support students in navigating both their gender exploration and associated mental health challenges. The heightened risk of concurrent mental health issues further complicates the experience for students with gender dysphoria [61–63], seen in several previous studies in the Nordic countries and in the US [8, 10, 11], issues which several systematic review express uncertainties in if puberty blockers help relieve on gender dysphoria [16–21].

The school nurses in the present study note that the school environment can be both inclusive and non-inclusive. In previous research [55], school nurses emphasise that schools should serve as spaces that promote well-being and provide opportunities for productivity; including students facing mental health challenges. These key aspects of the school nurse role in Swedish schools [37]. Some of the school nurses described how students with gender dysphoria could express themselves authentically at school, without the risk of facing derogatory comments. The nurses perceive the students’ peers as being generally accepting of their gender dysphoria. The school nurses in the present study highlight the absence of gender-neutral changing rooms. Previous research has shown that LGBTQ+ students often perceive changing rooms as spaces where they are exposed, which increases the risk of bullying and harassment [64]. The school nurses expressed a worry of doing something that could harm the students with gender dysphoria. Other research [30] suggests that adopting an open and accepting approach toward diversity can be beneficial when interacting with students in general. Previous research with school staff has claimed that transgender individual’s perceived well-being is strongly influenced by support from their family, friends. Furthermore, their well-being was influenced by a sense of safety within the school environment [24]. In previous research in England, Scotland, Wales and Australia [13, 14] transgender children, adolescents and their parents expressed how being able to live in accordance with one’s own gender identity and receiving social support and gender affirming care create sense of being confirmed in one’s identity. In contrast, other research with parents to children with gender dysphoria perceived their child to distrust non-transgender persons, trying to isolate themselves from family and non-transgender friends [12], with a systematic review showing that there was no difference in mental health outcomes for children who socially transitioned gender [15].

The present study reveals that some school nurses experience uncertainty regarding the issue of gender dysphoria. This uncertainty could be attributed to a lack of knowledge and limited experience with gender dysphoria. None of them seemed aware of the Guideline for Care of Children and Adolescents with Gender Dysphoria [6], which highlights how care for children and adolescents with gender dysphoria is constrained by limited accessibility and insufficient evidence on treatment outcomes. A knowledge of, and reliance on the guideline may aid school nurses in how to support students who experience gender dysphoria.

## Implications for practice and research

A key practical implication of this study is the need for targeted education, training, and competence development for school nurses, particularly regarding gender identities, gender dysphoria, and mental health, as well as the connection between them. Strengthening school nurses’ professional competence can reduce the burden placed on students to explain or advocate for themselves and can enhance health promotion efforts within the school setting.

Future research should further explore students’ perspectives on the school nurse’s role and examine how health dialogues can be adapted to better address gender identity–related issues, thereby contributing to more inclusive, supportive, and equitable school environments.

## Conclusion

The results demonstrate how when used intentionally, the health dialogue and survey can serve as a structured yet flexible tool for early identification, trust-building, and individualized support for students with gender dysphoria. Furthermore, the findings emphasize the school nurse’s role as a guide in inclusive school health promotion, including adapting the school environment to better support gender-diverse students. It also shows where the school nurses role is, and its limits. The results suggest that accessibility, openness, and a direct dialogue with the students with gender dysphoria can partially mitigate the challenge school nurses experience in their lack of knowledge and competence. The school nurses expressed a connection between gender dysphoria and associated comorbidities, especially mental health issues, underscoring the importance of school nurses’ awareness and preparedness to identify and respond to students’ needs.

## Electronic supplementary material

Below is the link to the electronic supplementary material.

Supplementary Material 1

## Data Availability

The datasets analyzed during the current study are not publicly available due to ethical principles but data not comprising confidential information are available from the corresponding author on reasonable request.
