# Supplementary material for: A qualitative content analysis study on Swedish school nurses’ experiences of meeting students with gender dysphoria
Source: BMC Nurs. 2026 May 26;25:485. doi: 10.1186/s12912-026-04801-x (PMC13214186; doi:10.1186/s12912-026-04801-x)
Supplement: Supplementary file 1 — Supplementary Material 1 [file 12912_2026_4801_MOESM1_ESM.pdf]

Supplementary file 1 – interview guide

<u>Demographic questions:</u> age, years of working as a nurse, master/specialist education, previous work life experience, description of the school they are currently working at

Interview questions

- What do you think of when hearing the term 'gender dysphoria'?
- Tell me about your experience with meeting students with gender dysphoria
- What do you think is important to think about when meeting and communicating with students with gender dysphoria?
- What challenges have you met when working within the school with students with gender dysphoria?
- What support have you observed that students with gender dysphoria have in their life?
- What support do you have from your employer to support these students?
- How do you collaborate with other professions in the school to support these students?
- Do you have anything else you would like to add that you have yet to tell me?
    - Possible follow up questions for all interview questions:
        - Coud you elaborate?
        - What would be an example of that?
        - Could you tell me more about that?
